# Overview: referrals for genetic evaluation from child psychiatrists

**DOI:** 10.1186/s13034-016-0095-6

**Published:** 2016-03-28

**Authors:** Katharine R. Press, Laura Wieczorek, Julie Hoover-Fong, Joann Bodurtha, Lynn Taylor

**Affiliations:** 733 North Broadway, Suite 137-Office of Student Affairs, Edward D. Miller Research Building, Baltimore, MD 21205 USA; Bloomberg Children’s Center, 12th Floor, Room 12316, 1800 Orleans Street, Baltimore, MD 21287 USA; Blalock 1008 Medical Genetics, 600 North Wolfe Street, Baltimore, MD 21287 USA; Bloomberg Children’s Center, 12th Floor, Room 12352, 1800 Orleans Street, Baltimore, MD 21287 USA

**Keywords:** Diagnosis, Genetics, Child psychiatry

## Abstract

A growing multitude of known genetic diagnoses can result in presentation to child psychiatry. For numerous reasons, it is important to identify a genetic etiology in child psychiatry patients when it is present. Genetic diagnoses can guide treatment and enable access to specialized clinics and appropriate screening measures. They can also allow for genetic counseling for the patient and family. A better understanding of etiology with a named diagnosis can itself be of great value to many patients and families; prognostic information can be empowering. Since patients with genetic conditions may present to psychiatric care in diverse ways, child psychiatrists must decide who to refer for genetic evaluation. Here we create a table to provide a framework of concerning/notable history and exam features that a practicing child psychiatrist may encounter that should prompt one to consider whether a larger, unifying genetic diagnosis is at hand. We hope this framework will facilitate referral of child psychiatry patients to genetics so that more patients can benefit from an appropriate diagnosis.

## Background

A growing multitude of known genetic diagnoses can result in presentation to child psychiatry. The prevalence of genetic diagnoses among child psychiatry patients is best studied for autism, where 10–20 % of cases have a diagnosable genetic cause [e.g. Fragile X (FXS)] [1–6]. There is little information about the prevalence of genetic conditions in child psychiatry patients more generally. There are a large number of different genetic conditions that may lead to psychiatric presentation in a child. A December 2015 search of the Online Mendelian Inheritance in Man database (OMIM.org^®^), a catalog of genetic conditions, reveals 42 genetic etiologic associations for both psychiatric symptoms and autism. As genetic understanding continues to progress, the overlap with child psychiatry grows and it becomes increasingly important for child psychiatrists to recognize signs of a possible underlying genetic diagnoses [7].

In some instances, a genetic condition may have a typical psychiatric presentation and psychiatric symptoms may appear isolated. In such cases, presentation may be indistinguishable from that of a typical psychiatric patient whose disease is polygenic and multifactorial and genetic diagnosis is more difficult [8]. However, in many cases a genetic etiology may be suggested by features of history or physical exam. If child psychiatrists are aware of red flags signifying possible genetic condition, more patients can be appropriately diagnosed.

This article addresses two questions: Why is it important to diagnose genetic conditions in child psychiatry patients? What should prompt a child psychiatrist to request a genetic consult for a patient?

## Main text

### Benefits of diagnosing genetic conditions

Identifying a genetic etiology in child psychiatry patients has many benefits [9]. Diagnosis of specific genetic conditions can guide treatment and allow access to specialized healthcare. For example, depression may herald Wilson disease, which can be effectively treated with chelators [10]. In metabolic disorders with psychiatric symptoms, a diagnosis can allow for appropriate management to avoid metabolic decompensations, which worsen both psychiatric and somatic symptoms [8]. In children with FXS, hyperactivity responds particularly well to methylphenidate. Hyperactivity is more responsive to methylphenidate as part of FXS versus part of autism spectrum disorder or intellectual disability, or possibly even non-specific ADHD [3]. When diagnosed, these patients can be put directly on methylphenidate and avoid many medication trials, which carry associated risks. Furthermore, because psychiatric symptoms may arise years before more specific organic signs, certain treatments may be more effective at the ‘psychiatric stage’ before occurrence of irreversible lesions. This is particularly true for metabolic disorders [11]. Genetic diagnosis also enables access to specialized clinics with greater understanding of and experience with these complex presentations [12]. There are a growing number of condition-specific and neurogenetic clinics that may be very beneficial to patients. Because wait times to appointments can be challenging, other consultation strategies (e.g. telehealth) may be considered.

Additionally, some genetic conditions are associated with known medical complications and may require routine screening or further medical work-up. For example, a patient presenting with autism spectrum disorder and macrocephaly who is found to have a PTEN mutation can then be enrolled in a cancer screening protocol. Further, diagnosis can allow for appropriate genetic counseling for the patient and family [12, 13].

Finally, a better understanding of etiology with a named diagnosis may itself be of great value to many. Most individuals would rather know they have a serious disease than continue without a diagnosis [14]. Diagnosis allows for improved understanding, educational planning, and social support, and peer networking [15, 16]. Prognostic information can be empowering.

### Indications for genetic consultation

It is not practical to conduct a genetic workup on all patients, so child psychiatrists must decide whom to refer for genetic evaluation [1]. Child psychiatrists are in an important position to diagnose genetic conditions, as they often see children for whom a diagnosis has been elusive, who have unusual presentations, or who have been difficult to treat.

Patients with genetic conditions may present to psychiatric care in diverse ways. Table 1 shows genetic conditions that may in rare cases account for typical child psychiatry presentations. Psychiatric symptoms may be apparently isolated, making genetic diagnosis difficult. However, often a careful history and physical provides hints to a genetic diagnosis.

**Table 1:** Genetic syndromes may in rare cases manifest in more general child psychiatry presentations

Psychiatric presentation	Example of genetic syndrome that may result in this presentation
Anxiety	22q11 deletion [17], Fragile X syndrome [18], Williams syndrome [19]
Acute psychosis	Adrenoleukodystrophy [20], Porphyria [21], Niemann-Pick [21], 22q11 deletion [17]
Conduct disorder/poor judgment/anger	Monoamine oxidase A deficiency [22]
Hyperactivity	Turner syndrome [23], Fragile X syndrome [3]
Depression	Wilson disease [24]

Some unusual psychiatric presentations or behaviors may suggest a particular genetic etiology. Skin picking may occur in Prader-Willi syndrome [25] while severe disruptions of sleep and nail pulling suggest Smith-Magenis syndrome [26]. In many cases, non-psychiatric features of the history or exam will provide clues to genetic etiology.

The purpose of Table 2 is to present a framework of notable history and exam features that should prompt a child psychiatrist to consider whether there is a larger, unifying genetic diagnosis at hand. All of these features are recognized as markers in pediatric genetic practice, however here the goal is to isolate the flags most likely to present in a child psychiatry practice [27]. This table is just a starting point and should not be considered exhaustive.

**Table 2:** Red flags for a possible genetic diagnosis in child psychiatry patients

Red flag	Explanation	Examples of genetic conditions that may include specific psychiatric components
Medical history		
Autism spectrum disorder	Genetic causes can be identified in 10–20 % of autism patients [1–6]	Rett syndrome PTEN mutations Tuberous sclerosis Chromosomal copy number variants
Intellectual disability or global developmental delay	While these findings may be purely developmental, they should be investigated further when the findings seem out of proportion to the level of developmental delay	Rett syndrome fragile X
Psychiatric symptoms worsening with conditions leading to increased protein catabolism, such as fever, surgery, or prolonged fasting	May indicate metabolic dysfunction	
Unusually severe presentation or prolonged recovery after minor illness		Acute intermittent porphyria
Cyclic or recurrent vomiting, particularly with protein intake		Organic acidemias Inborn error in pyruvate metabolism
Poor or atypical treatment response to medications or behavioral interventions	May indicate an alternate or additional diagnosis to explain nonstandard response/components	Prader-Willi syndrome
Severely disrupted sleep		Smith-Magenis syndrome
Self-injurious behavior or skin picking		Lesch-Nyhan syndrome, Prader-Willi syndrome
Family history		
Significant family history of psychiatric conditions	Though purely psychiatric conditions may also follow a familial pattern, this could indicate an underlying genetic diagnosis	22q11 deletion syndrome
Significant family history of neurologic regression or progressive neurologic disorders	May represent certain autosomal dominant traits for which first symptoms are sometimes psychiatric	Huntington disease Spinocerebellar ataxias
Family history of relatives with intellectual disabilities or many with learning disabilities	Intellectual disability and learning disabilities are part of many genetic syndromes that also have psychiatric features	Fragile X syndrome
Born to a parent with a known cytogenetic abnormality (e.g. balanced translocation) or recurrent pregnancy loss	Translocations may become unbalanced in subsequent generations causing a variety of presentations including psychiatric disease and pregnancy loss	Unbalanced chromosomal complement
Physical exam		
Dysmorphic features that are not familial	May be caused by genetic syndrome	22q11 deletion syndrome Fragile X Submicroscopic chromosomal deletions and duplications Lysosomal storage diseases
Single major or multiple minor and/or major physical anomalies		Branchio-oto-renal syndrome Holt Oram syndrome Chromosomal deletions or deletions
Striking inability to learn after many well-controlled trials	May suggest cortical dysfunction	Fragile X
Hepatosplenomegaly	These findings would not be explained by a psychiatric diagnosis alone. In conjunction with psychiatric symptoms, these findings may suggest a unifying genetic diagnosis.	Gaucher disease Niemann-Pick disease Mucopolysaccharidoses
Unusual body odor		Glutaric aciduria type II Phenylketonuria Isovaleric academia Maple syrup urine disease
Unusual dermatologic findings: multiple types of lesions, six or more café au lait macules >1.5 cm in diameter, multiple lipomas, albinism		Neurofibromatosis type I Cowden syndrome Hermansky-Pudlak syndrome
Unexplained neurologic findings Intractable seizures Hypertonia or hypotonia Peripheral neuropathy Myopathy Progressive ataxia		Gaucher disease Neuronal ceroid lipofuscinosis Mitochondrial disorders Inborn errors of metabolism including leukodystrophies Prader-Willi syndrome Angelman syndrome Charcot-Marie-Tooth disease Spinocerebellar ataxias
Evidence of a connective tissue disorder Joint laxity Poor wound healing Marfanoid habitus		Homocystinuria Ehlers Danlos syndrome
Unexplained lab anomalies Acidosis Persistent hypoglycemia Adrenal insufficiency		Organic acidemias Beckwith-Wiedemann syndrome Adrenoleukodystrophy
Abnormal brain MRI findings		Tuberous sclerosis Leukodystrophies Spinocerebellar ataxias, Alexander disease
Microcephaly		Fetal alcohol syndrome, Williams syndrome
Failure to thrive or short stature	While psychiatric illness may lead to growth abnormalities, these abnormalities should be evaluated further when they cannot be fully explained by psychiatric disease	Various chromosomal conditions

If one or more of these red flags are present, a child psychiatrist should consider requesting a genetic consultation. Whenever possible, genetic tests should be ordered in conjunction with a genetic team. On a practical level, determining which genetic tests to order can be confusing and outside the usual practice of most child psychiatrists. Even more important, many child psychiatrists are not comfortable explaining the intricacies of genetic testing and lack the infrastructure to deal with ramifications of positive results [28]. Informed consent must include discussion of the risks of genetic testing, which include harms associated with resultant treatments and incidental findings that can be life changing or psychologically disruptive [29]. There may be particular cases in which a child psychiatrist has experience with or training relevant to a certain genetic test and feels comfortable ordering this test, so this remains an individual call. However, in most cases it is best to involve a genetic team and employ their associated infrastructure. It is our hope that this table will help child psychiatrists communicate with genetic teams by allowing them to pinpoint the red flags that led them to consider a genetic etiology.

## Conclusions

It is important to identify a genetic etiology in child psychiatry patients when it is present, as it has implications for treatment and counseling. Patients with genetic conditions may have unusual psychiatric symptoms or other abnormal history and physical findings. Here we present a table of red flags that a practicing child psychiatrist may identify, which could indicate an underlying genetic diagnosis. We hope this table will inspire child psychiatrists to think about genetic possibilities in their patient populations and make referrals for genetic evaluation when appropriate. Future work will be needed to validate this table in practice. However, being aware of these the flags has immediate utility in the clinical practice of child psychiatrists. To highlight how simple practice changes may have a big impact for diagnosing conditions, we provide a short list of tips and resources for making a genetic diagnosis in child psychiatry (Table 3). We hope that this commentary will encourage child psychiatrists to think about their growing overlap with the field of genetics. Recognition of the importance of genetic diagnoses in child psychiatry patients may stimulate more research into the prevalence of genetic disease, effective methods of screening and diagnosis, and strategies for treatment and management for these patients.

**Table 3:** Short list of tips and resources for making genetic diagnoses in child psychiatry

1.	Take a family history
2.	Measure and plot head circumference percentiles
3.	Inspect the skin
4.	Take note of dysmorphic features
5.	Know your genetics referral colleagues
6.	Short list of genetic resources GeneReviews^®^: http://www.genereviews.org/ Online Mendelian Inheritance in Man (OMIM)^®^: http://www.omim.org/ Genetics Home Reference: http://ghr.nlm.nih.gov/
